# Comparative Analysis of Transcriptomes to Identify Genes Associated with Fruit Size in the Early Stage of Fruit Development in *Pyrus pyrifolia*

**DOI:** 10.3390/ijms19082342

**Published:** 2018-08-09

**Authors:** Shuang Jiang, Haishan An, Jun Luo, Xiaoqing Wang, Chunhui Shi, Fanjie Xu

**Affiliations:** Forestry and Pomology Research Institute, Shanghai Key Lab of Protected Horticultural Technology, Shanghai Academy of Agricultural Sciences, 1000 Jinqi Road, Fengxian District, Shanghai 201403, China; jiangshuang@saas.sh.cn (S.J.); anhaishan530@163.com (H.A.); wxqshirley666@163.com (X.W.); shichunhui6666@163.com (C.S.); funjx@163.com (F.X.)

**Keywords:** fruit size, *Pyrus*, cell division, development, transcript

## Abstract

Pear (*Pyrus* L.) is an important commercial fruit in the world. The fruit size is one of the important characters in fruit quality. The previous research reported that the fruit size of pear was mainly caused by the number of cell in about 40 days after blossom (DAB) in nature. However, studies about the mechanisms underlying cell division in young fruit development are very limited in pear. Two pear accessions codenamed ‘GH59B’ with big fruit and ‘GH81S’ with small fruit in three stages were sampled and the RNA-seq high-throughput sequencing was used to evaluate changes of gene transcription levels in the early stage of fruit development. The difference of cell size among two samples was little in 40 DAB, implying that the difference of the fruit size was caused by the number of the cell. More than 274,517,982 high quality reads from six libraries of fruit development were sequenced. A total of 797 differentially expressed genes (DEGs) were identified. Three cytokinin dehydrogenase genes and two gibberellin 2-beta-dioxygenase gene were identified in the Kyoto Encyclopedia of Genes and Genomes (KEGG) pathways related to zeatin and gibberellin. Their expression was upregulated at 20 DAB in ‘GH81S’ and at 30 DAB in ‘GH59B’, suggesting that the small fruit size might be related to the early degradation of cytokinin and gibberellin inducing a short period of cell division. A total of 38 DEGs of transcription factors were found and 23 DEGs including *NAC*, *ERF* and *bHLH* transcription factors were highly related with cytokinin dehydrogenase and gibberellin dioxygenase genes. Altogether, the results of the present study provide information from a comprehensive gene expression analysis and insight into the molecular mechanism underlying the difference of fruit size in *Pyrus pyrifolia*.

## 1. Introduction

Fruit size is not only a very important appearance quality but also strongly associated with the commercial value. The environmental factors, chemicals and internal heredity could regulate fruit size. For example, thinning and adequate nutrition can increase fruit size [1,2]. Benzyladenine, forchlorfenuron (CPPU) and gibberellin treatments could increase fruit weight and diameter [3,4]. The genetic background could also influence fruit size. Under the same growth conditions, some pear cultivars in *Pyrus pyrifolia* have large fruits (such as ‘Housui’) and some wild pear accessions in *P. betulaefolia* have small fruits. Fruit size is determined by the number of cells and cell size during fruit development. In apple, fruit size was positively correlated with cortex cell number [5]. In melon, that fruit size is also determined by the amount of cell proliferation in the early stage of fruit development [6]. In sweet cherry, the average cell numbers were significantly different between cultivars with big and small fruit, indicating that flesh cell number is the major contributor to differences in fruit size between cultivars [7]. These reports indicated the difference in cell numbers affecting fruit size. Some other studies have shown that both of cell number and cell size induce fruit size difference. In *Malus floribunda*, whose fruit size is one of the smallest among *Malus*, exhibited absence of cell proliferation throughout fruit ontogeny. Furthermore, the rate of cell enlargement, which was almost uniform until the mature stage in other species, slowed down from about 45 days after full blossom, resulting in a cell size approximately half that of the other cultivars [8]. The carpel/floral-tube size was enhanced in ‘Grand Gala’, which was a large fruit size spontaneous mutant of ‘Gala’. It was associated with cell number and size [9]. 

The growth curve of the pear is close to the S type. The cell proliferation increased significantly in the 3–4 weeks after pollination, which was like apple [10]. The growth of the fruit was mainly manifested by the increase in the number of cells in this period. The late maturing varieties have larger fruit types than those of early maturing varieties in Japanese pears and the difference in fruit size among varieties was mainly determined by the number of cells, not the size of the cells [11]. The late-ripening varieties were often larger than those of early maturing varieties. The reason is mainly related to the extension stage of cell division.

A lot of genes were related to cell number and cell size [12]. In tomato, two developmental timing of genes of fruit weight 2.2 (*FW2.2*) and *FASCIATED* known to affect tomato fruit size and shape were reported [13]. The gene *FW2.2* regulates the size of fruit by regulating the proliferation of early growth and development of the fruit [14,15]. The gene *FASCIATED* encodes a *YABBY-like* transcription factor, which regulates the size of tomato fruit by regulating the number of carpels [16]. Silencing of the *DELLA1* gene in tomato produced very similar vegetative and reproductive phenotypes to those described for gibberellin 20-beta-dioxygenase 1 (*GA20ox1*) overexpressing plants [17], as fruits were facultative parthenocarpic, smaller in size and elongated in shape. Some other genes promote cell proliferation and cell enlargement to regulate the growth and size of plant tissue [18,19]. In *Arabidopsis thaliana*, the transcription factor AINTEGUMENTA (*ANT*) can affect the end of cell proliferation and it is a key gene to regulate the size of tissue [20]. The auxin-inducible gene *ARGOS* can regulate the size of the lateral tissue and prolong the expression of *ANT* [21]. *KLUH*, a cytochrome P450 gene, participates in the formation of the RNA polymerase complex. The overexpression of *KLUH* leads to larger organs with more cells [22]. The *JAGGED* gene encodes a zinc finger protein that promotes leaf tissue development [23]. *BIG BROTHER*, an *E3* ubiquitin ligase, is a repressor of plant organ growth and alter organ size [24]. In addition. The proliferation of plant cells is also regulated by the cell cycle. In *Arabidopsis*, the cell cycle is regulated by cyclin, cyclin dependent kinase (*CDKs*), cyclin kinase inhibitor (*ICK/KRPs*) and *E2F* transcription factor [25]. The overexpression of *CDKB1;1* can reduce the number of cells [26] and a small amount of cell cycle protein kinase inhibitor *KRP2* can reduce cell production rate, decrease leaf area and change leaf shape [27].

In *P. pyrifolia*, the fruit size was related to the cell number. The cell division occurred in the early stage in the development of fruit. Therefore, studies on the variation of fruit size in young fruit stage might be more helpful for understanding different fruit size in *Pyrus*. In this study, we selected two pear accessions with different fruit size in hybrid F1 generation from ‘Gold Nijiss Qiki’ and ‘Housui’ and collected samples of young fruits at 10, 20 and 30 days after blossom. The transcriptome was investigated in fruit development to reveal the molecular mechanism of cell division. We sequenced six cDNA libraries using Illumina deep-sequencing technology. The gene expression profiles will provide valuable resources for the identification of pear genes involved in fruit size difference.

## 2. Results

### 2.1. The Fruit Weight and Histological Sections of ‘GH59B’ and ‘GH81S’ in Forty Days after Blossom

**Figure 1:**
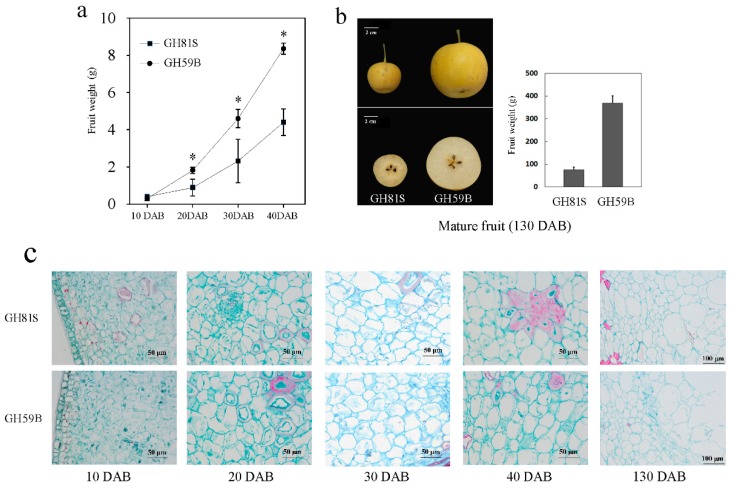
Development of the fruit of ‘GH81S’ and ‘GH59B’ in 40 days after blossom. (**a**) Fruit weight. The asterisks represent significant differences determined by Student’s *t* test at *p* < 0.05. (**b**) The mature fruit, (**c**) Histological sections of young fruit.

**Figure 2:**
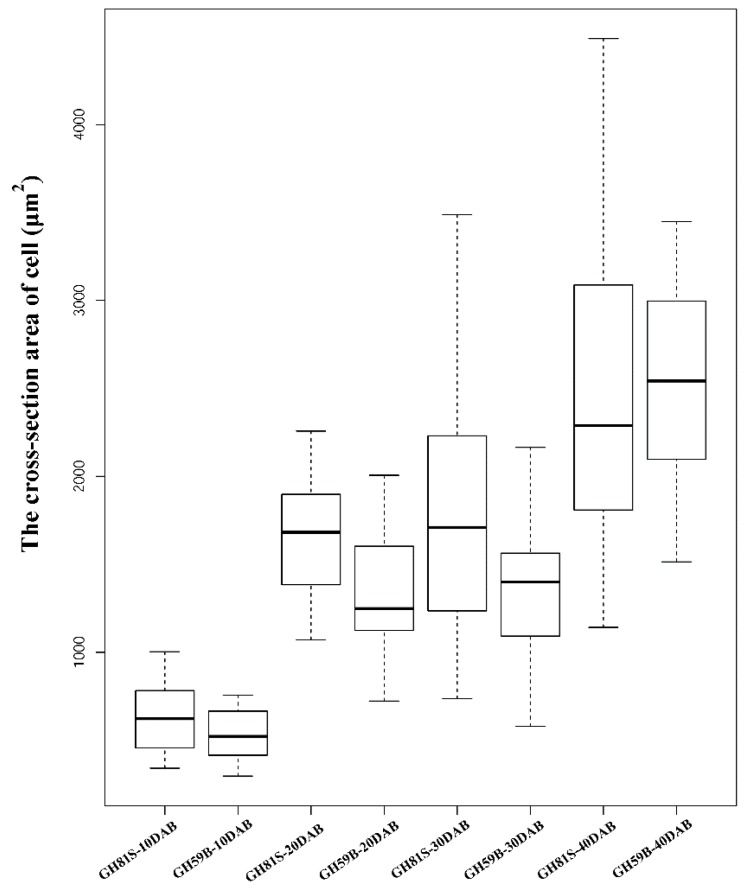
The cross-section area of cell size of ‘GH81S’ and ‘GH59B’ in 40 days after blossom.

The fruit weight of ‘GH59B’ and ‘GH81S’ were measured at 10, 20, 30, 40 and 130 DAB. The weight of both samples was increased in the development of fruit (Figure 1a). At 10 DAB, the weight between ‘GH59B’ and ‘GH81S’ had no obvious difference. The differences were appeared before 20 DAB, right after 10 DAB and expanded gradually. The mature fruit weight of ‘GH59B’ and ‘GH81S’ were 368.4 g and 75.5 g at 130 DAB, respectively (Figure 1b). By visual inspection, the anatomical structures of the pulp cell showed little difference in the cell size of two samples (Figure 1c). The cross-section area of ‘GH59B’ and ‘GH81S’ were calculated. The cell size was increased largely in both of two samples in all stage of fruit development (Figure 2). The cell size has grown about twice between 10 DAB and 20 DAB. The median of cell size in ‘GH81S’ was larger than in ‘GH59B’ in early three stage of fruit development. At 40 DAB, the cell size of ‘GH59B’ was larger than ‘GH81S.’ The difference in cell size among the two samples was little. Therefore, we implied that the difference of the fruit size was caused by the number of cell in early fruit development. At 130 DAB, the cell size varied largely in ‘GH59B’ and ‘GH81S.’ The cross-section area of the cell was difficult to calculate. Some very large cells were found (Figure 1c), which might be related with the subsequent rapid fruit growth.

### 2.2. Library Construction, Sequencing and Differentially Expressed Genes (DEGs) Identified Using RNA-Seq

The fruit of ‘GH59B’ and ‘GH81S’ were sampled at 10, 20 and 30 DAB and were subjected to total RNA extraction and an RNA-seq analysis. High-throughput sequencing generated 47.46–52.40 million (M) 150 bp paired-end reads from each library (Table 1). After a stringent quality filtering process, 41.18 Gb of clean data (90.86% of the raw data) were obtained, with a Q30 percentage ≥94%. The counts of clean reads per library ranged from 43.56 to 46.79 M (Table 1). Reads were mapped to the reference genome sequence of Chinese white pear ‘Dangshansuli’ (*Pyrus pyrifolia* White pear group) [28]. The percentages of mapped reads were similar among the 6 libraries (68.7–70.4%) (Table 1). 

**Figure 3:**
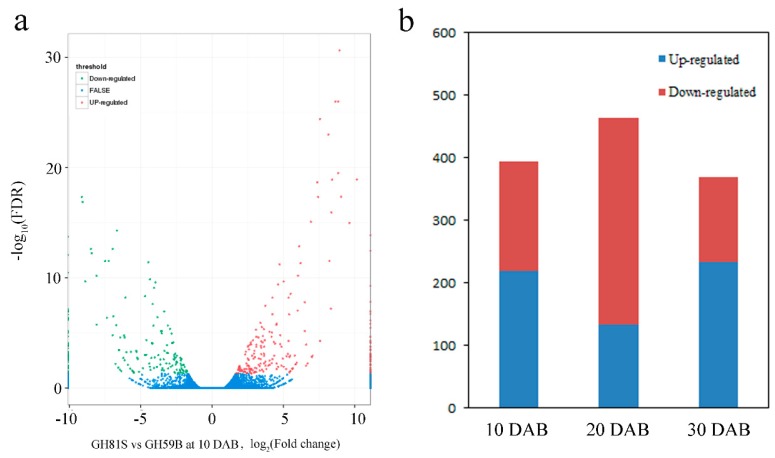
Statistics of differently expressed genes. (**a**) Significantly up- or downregulated genes using the threshold of FDR ≤ 0.001 and log_2_Ratio ≥ 1 in GH81S vs. GH59B at 10 DAB. (**b**) Number of upregulated and downregulated transcripts in three stage of fruit development.

**Figure 4:**
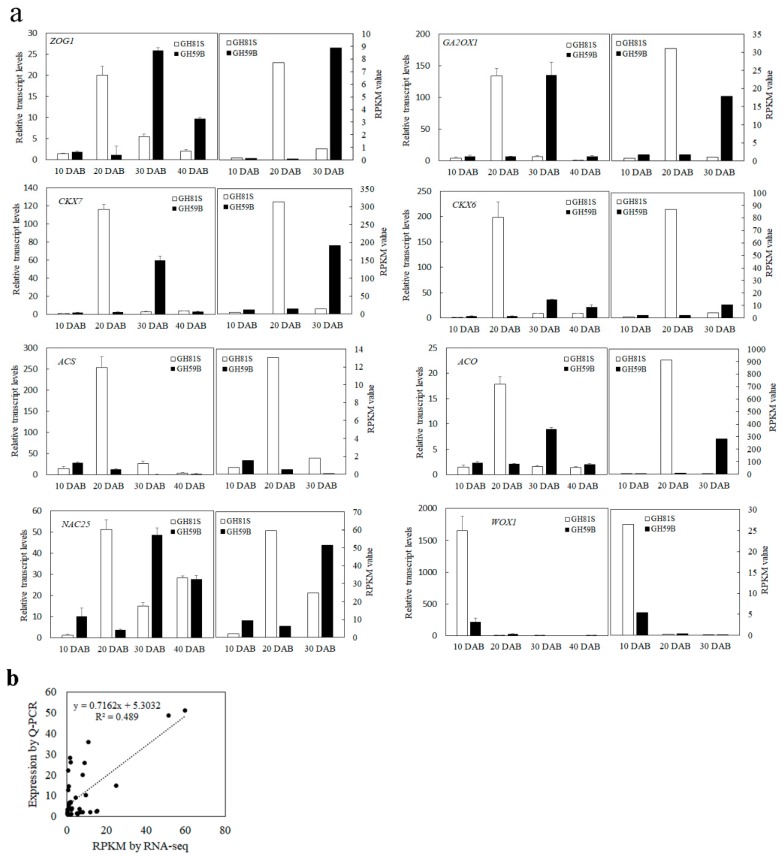
The expression of eight genes in fruit of ‘GH81S’ and ‘GH59B.’ (**a**) Q-PCR validation of differential gene expression. (**b**) Coefficient analysis between the gene expression ratios obtained from RNA-seq and Q-PCR data.

The genes with reads per kilobase of transcript per million mapped reads (RPKM) value of six samples were calculated. Differences in gene expression in six samples were examined using the threshold of FDR ≤ 0.001 and |log2Ratio| ≥ 1. The DEGs were identified by pairwise comparisons of the libraries from ‘GH59B’ and ‘GH81S’; that is, GH81S-10DAB vs. GH59B-10DAB (Figure 3a). For each stage; 393 DEGs were detected at 10 DAB. At 20 DAB; 463 DEGs were identified; which is more than in 10 DAB and 30 DAB (368 DEGs) (Figure 3b). After merging the DEGs in three stages; 797 DEGs were found in the early fruit development in pear; which was investigated further (Table S1). Eight genes were selected to have their transcript levels measured by Q-PCR; including Zeatin O-glucosyltransferase 1 (*ZOG1*), cytokinin dehydrogenase 6 (*CKX6*), cytokinin dehydrogenase 7 (*CKX7*), Gibberellin 2-beta-dioxygenase 1 (*GA2OX1*), 1-aminocyclopropane-1-carboxylate synthase (*ACS*), 1-aminocyclopropane-1-carboxylate oxidase (*ACO*), *NAC25* transcription factor; and WUSCHEL-related homeobox 1 (*WOX1*). The differential expression trends detected by Q-PCR and RNA-seq were largely consistent (Figure 4), indicating the reliability of the RNA-seq results.

### 2.3. DEGs Involved in the KEGG Pathways Related to Zeatin and Gibberellin

All DEGs were mapped with the KEGG database to identify the significant enrichment of pathways. A total of 82, 89 and 80 pathways were found at 10, 20 and 30 DAB respectively. The size variety of ‘GH59B’ and ‘GH81S’ was speculated to be caused by the cell number and the cell number in fruit development was related with zeatin and gibberellin in the previous research. The KEGG pathways of zeatin biosynthesis (ko00908), gibberellin related diterpenoid biosynthesis (ko00904) and the plant hormone signal transduction of zeatin and gibberellin (ko04075) were investigated to scan the putative key genes. In all isolated DEGs, twenty-seven DEGs were found in three KEGG pathways (Table S2). Twelve DEGs were related to zeatin, of which eight DEGs in biosynthesis and four DEGs in the signal transduction were found respectively. In zeatin biosynthesis pathway, two genes of cytokinin dehydrogenase (*CKX7*, Pbr024972.1; *CKX6*, Pbr009698.1) and one gene of Zeatin O-glucosyltransferase 1 (*ZOG1*, Pbr002045.1) were isolated. Both of them were regarded as a main negative regulator in cytokinin metabolism in plants, irreversibly degrades cytokinins into adenine/adenosine moiety. In ‘GH81S’ with small fruits, these genes were upregulated at 20 DAB but they were upregulated at 30 DAB in ‘GH59B’ with big fruits (Figure 4). At 10 and 40 DAB, these genes maintained the low level of expression. Nine DEGs in diterpenoid biosynthesis and six DEGs in corresponding signal transduction were found. In gibberellin biosynthesis, Gibberellin 2-beta-dioxygenase 1 (*GA2OX1*) used bioactive GAs and their immediate precursors and converted them to inactivity forms. In our result, two genes of *GA2OX1* (Pbr025274.1) and *GA2OX8* (Pbr009976.1) were isolated. Their expression was like cytokinin dehydrogenase genes. They were upregulated at 20 DAB in ‘GH81S’ and at 30 DAB in ‘GH59B’.

### 2.4. The Annotation of Structural Genes Related to Fruit Development

In all isolated DEGs, function unknown and low expression genes were removed firstly and then the genes not related to fruit development were deleted, such as resistance protein and leucine-rich repeat receptor (Table S1). Structural genes and transcription factors were analyzed separately. Twenty-four structural DEGs were identified (Table S3). Three genes (Pbr009641.1, Pbr016511.2 and Pbr040203.1) were annotated to xyloglucan endotransglucosylase (*XET*), which was induced by brassinosteroid and improved cell division. Pbr016511.2 was upregulated in all stage in ‘GH59B’ and Pbr009641.1 was upregulated at 20 DAB in ‘GH59B’. Pbr040203.1 was upregulated at 20 DAB in ‘GH81S’ and 30 DAB in ‘GH59B.’ Four genes were related to the pathway of ethylene biosynthesis and promote the synthesis of ethylene. Pbr002233.1 was annotated to 1-aminocyclopropane-1-carboxylate synthase (*ACS*). Three genes (Pbr023059.1, Pbr031954.1 and Pbr015589.1) were annotated to 1-aminocyclopropane-1-carboxylate oxidase (*ACO*). Except for Pbr015589.1, the expression of the other three genes were significantly upregulated at 20 DAB in ‘GH81S’ and at 30 DAB in ‘GH59B,’ which was similar with cytokinin dehydrogenase genes. One gene of WUSCHEL-related homeobox (*WOX1*) was isolated, which plays important roles in the maintenance and proliferation of the stem cell niche in the shoot apical meristem. It was up-regulated at 10 DAB in both of ‘GH81S’ and ‘GH59B’ and the gene expression in ‘GH81S’ was higher than in ‘GH59B’. Two genes (Pbr027542.1 and Pbr016230.1) were related to the auxin-repressed protein. Their expression was up-regulated at 10 and 30 DAB in ‘GH59B.’ Fourteen DEGs were annotated to the cytochrome P450. Pbr009519.1 (*CYP90A1*) and Pbr030002.1 (*CYP734A1*) were involved in brassinosteroids inactivation and regulation of BRs homeostasis. Their expression was upregulated at 20 DAB in ‘GH81S’ and upregulate at 30 DAB in ‘GH59B.’ The gene of *CYP714* encodes gibberellin 13-oxidases that reduce gibberellin activity. Two genes of *CYP714A1* (Pbr002890.1) and *CYP714C2* (Pbr017314.1) were isolated and they were upregulated at 20 and 30 DAB in ‘GH81S,’ which might inhibit the cell growth. One gene of *CYP78A9* (Pbr018401.1) was identified, which was related to induce large and seedless fruit in *Arabidopsis*. The expression of *CYP78A9* was upregulated at 10 and 20 DAB in ‘GH59B’ but 30 DAB in ‘GH81S’. The function of the remaining nine genes from cytochrome P450 was unknown, which were not further analyzed.

### 2.5. The DEGs Related to Transcription Factors

**Figure 5:**
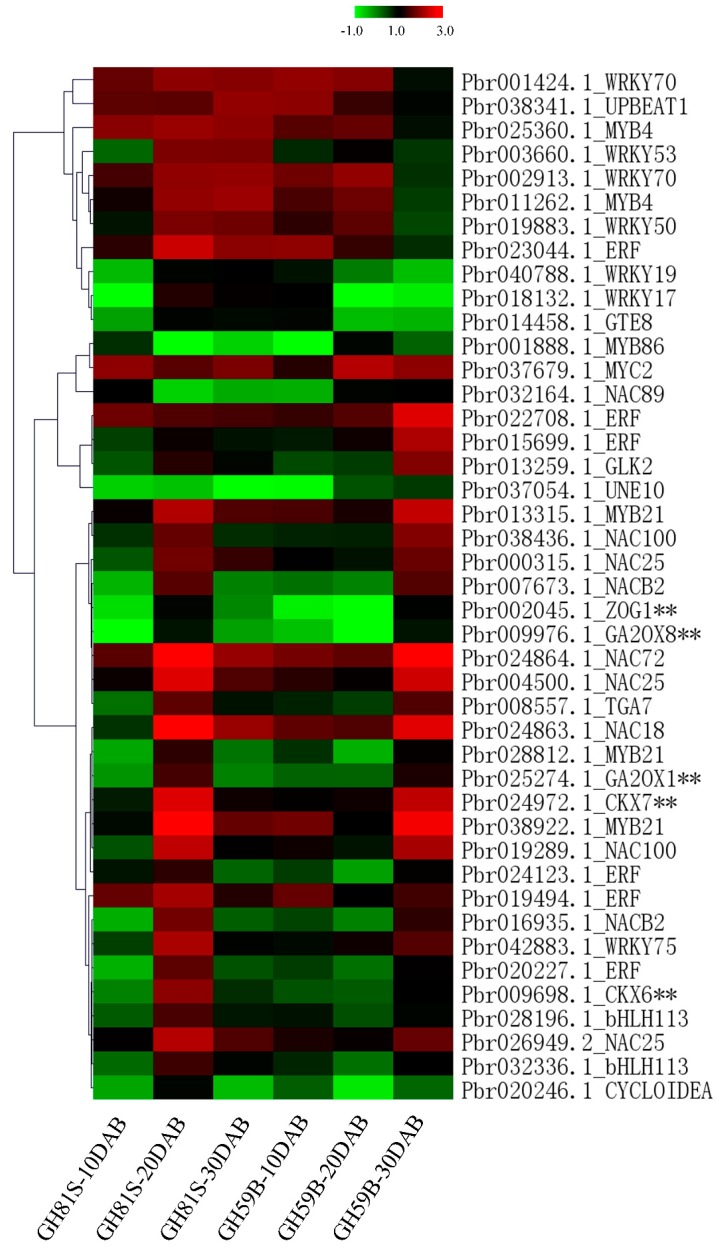
Heatmap of the expression of transcription factors.

Transcription factors play important roles in the regulatory network. A total of 38 DEGs were annotated to transcription factors (Table S4). All DEGs were clustered with cytokinin dehydrogenase and gibberellin dioxygenase genes to identify the putative upstream regulation genes (Figure 5). Group1 including 23 DEGs and Group 2 including 15 DEGs were clustered separately (Table S4). The expression of genes in Group 1 was highly related to cytokinin dehydrogenase and gibberellin dioxygenase genes. Nine genes were annotated to the *NAC* transcription factor, which involves plant growth and development, hormone regulation and response to the environmental stress. Four genes were ethylene responsive transcription factor (*ERF*). This factor regulates various physiological processes of plant growth and development. Lager number of members were found in *NAC* and *ERF* families implying that these families might participate in the development of fruit and determine the size of the fruit. Three *MYB21* and two *bHLH113* transcription factor were isolated. The remaining five transcription factors were annotated to *WRKY75*, *GLK2*, *UNE10*, *TGA7* and *CYCLOIDEA*. These transcription factors were rarely reported to regulate the fruit development.

### 2.6. Protein Protein Interaction (PPI) Network Analysis

**Figure 6:**
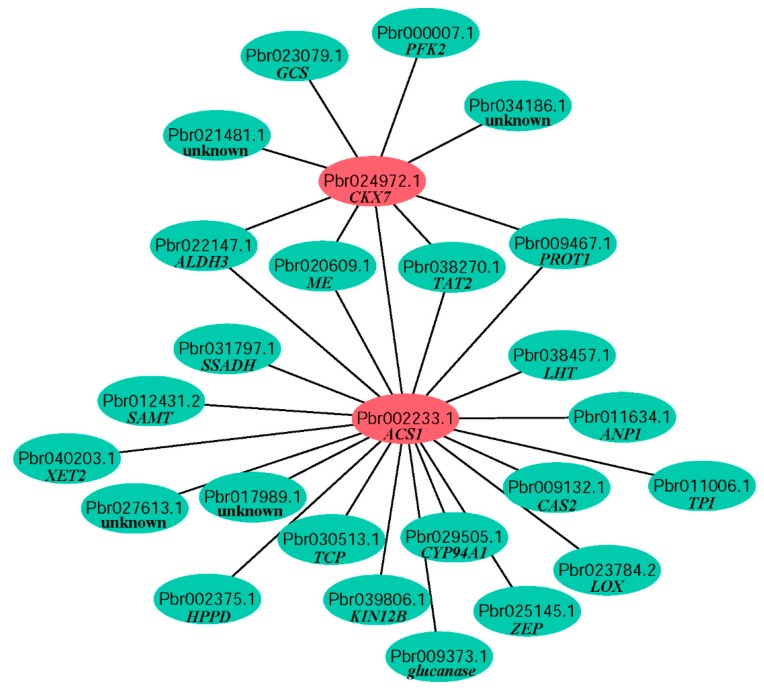
Protein protein interaction of CKX7 and ACS.

PPI network could identify putative direct physical interactions and indirect functional correlations between two proteins. All 797 DEGs were mapped with genes in PPI network from *Arabidopsis* in STRING database. A lot of networks in three stages were found and networks including genes with large differences between ‘GH81S’ and ‘GH59B’ were showed in Figure S1. Five genes of *ZOG1*, *CKX6*, *CKX7*, *GA2OX1* and *GA2OX8* in the previous result were searched the PPI network in all 797 DEGs. Only *CKX7* (Pbr024972.1) was found in a network and interacted with eight genes (Figure 6). One gene was annotated to *ACS*, suggesting that cytokinin dehydrogenase was related with ethylene synthesis. Although the *ACO*, one of rate-limiting enzyme of the ethylene biosynthetic pathway, was not found in the network with *CKX*, the expression profile of *ACO* was also consistent with *ACS* and *CKX*. Four genes were found to be related with *ACS* and *CKX7* and were annotated to aldehyde dehydrogenase 3 (*ALDH3*, Pbr022147.1), NAD-dependent malic enzyme (*ME*, Pbr020609.1), probable aminotransferase TAT2 (Pbr038270.1) and proline transporter 1 (*PROT1*, Pbr009467.1). The function of these four genes in fruit development was still unclear. The remaining two genes were glycine cleavage system H protein 3 (*GCS*, Pbr023079.1) and 6-phosphofructokinase 2 (*PFK2*, Pbr000007.1), which were not related with fruit development. In the network of *ACS*, 16 DEGs were found (Figure 6). Most of them were related to metabolic pathways and their function should be further analysis. 

## 3. Discussion

In pear breeding, breeders like to choose large fruit cultivars as parents to produce hybrids with big fruits. Therefore, the cultivars with big fruits have already accumulated many genetic loci inducing big fruit by artificial selection [12]. The loci might be different, suggesting that many genes could increase the fruit size. In previous studies, the cell division stage of large fruit pear is longer than that of small fruit pear at the young fruit development. The pear size was mainly related to the cell number, rather than the cell size [11]. The cell division was occurred in the development of young fruit, mostly in forty days after blossom. Therefore, we choose two pear accessions with a consistent phenological period from the hybrids of ‘Gold Nijiss Qiki’ and ‘Housui’ to minimize the genetic background differences in this study. The differences of fruit size between ‘GH59B’ with big fruit and ‘GH81S’ with small fruit were appeared at 20 DAB and expanded gradually to 30 DAB. The cross-section area of ‘GH59B’ and ‘GH81S’ showed that the cell size was enlarged in both of samples, suggesting that the cell size enlargement was one of the factors to increase the fruit weight. The cell size between ‘GH59B’ and ‘GH81S’ showed little difference in four stages of fruit development, which proved that the fruit size difference between ‘GH59B’ and ‘GH81S’ in young fruit development was induced by cell number. The same results were also found in melon and sweet cherry [6,7].

Since the fruit size was related to the cell division in the early stage of fruit development, the RNA-seq was used to identify the putative important genes in young fruit development. In this study, 797 DEGs were found in the comparison between ‘GH59B’ and ‘GH81S.’ A total of eighty-nine DEGs were identified to be related with pear fruit development. Twenty-seven of them were zeatin and gibberellin related genes. Twenty-four of them were other structural genes and thirty-eight of them were transcription factors. Spraying zeatin and gibberellin could increase fruit size in many crop plants, such as apple [29], peach [30] and pepper [31]. Therefore, the KEGG pathways of zeatin, gibberellin and corresponding signal transduction were firstly analyzed to isolate putative key genes. Five DEGs were identified. Three DEGs were related to cytokinin including one gene of *ZOG1* and two genes of *CKX*. *ZOG* inhibited the growth of rice. The overexpression of the *cZOG1* and *cZOG2* genes exhibited short shoot phenotypes, delay of leaf senescence and the decrease in crown root number [32]. *CKX* is an enzyme that degrades the cytokinin, which negatively regulates plant growth. Reduced expression of *Gn1a* (*OsCKX2*) in rice causes cytokinin accumulation in inflorescence meristems and increases the number of reproductive organs, resulting in enhanced grain yield (Ashikari, et al., 2005). In *Arabidopsis*, *ckx3* and *ckx5* double mutants form large inflorescences and floral meristems and contain supernumerary ovules, thus leading to an increase in seed set per silique [33]. In this study, the *ZOGT1*, *CKX6* and *CKX7* were upregulated at 20 DAB in ‘GH81S’ and at 30 DAB in ‘GH59B’, suggesting that the early expression of these genes might terminate the cell division of fruit in ‘GH81S.’ The Period of cell division in ‘GH59B’ might be longer than ‘GH81S.’ Two DEGs were related to gibberellin 2-beta-dioxygenase. Overexpression of the gibberellin 2-oxidase gene causes dwarf phenotypes in *Tricyrtis* sp. [34] and dwarfism and smaller flowers in *Nicotiana tabacum* [35]. The expression profile of *GA2OX1* and *GA2OX8* were consistent with *ZOG* and *CKX* in early pear fruit development, implying that these genes might play critical roles in cell division. The size of pear might be related to the temporal expression of these genes. Both of cytokinin and gibberellin might participate in the regulation of fruit development.

Beside cytokinin and gibberellin related genes, some other DEGs were identified. Two types of genes related to ethylene and brassinosteroids might involve in the fruit development. One gene of 1-aminocyclopropane-1-carboxylate synthase (*ACS*) and three genes of 1-aminocyclopropane-1-carboxylate oxidase (*ACO*) were identified. ACS is a rate-limiting enzyme in ethylene biosynthesis and ACO catalyzes the last step in the biosynthesis of ethylene [36]. In the present study, *ACS* (Pbr002233.1) and *ACO* (Pbr023059.1, Pbr031954.1 and Pbr015589.1) were upregulated at 20 DAB in ‘GH81S,’ suggesting that the ethylene might be synthesized in this period in ‘GH81S.’ In ‘GH59B,’ the gene of *ACS* was always expressed lowly but three genes of *ACO* were upregulated significantly at 30 DAB in ‘GH59B,’ implying that the synthesis of ethylene might be delayed in pear with big fruit. Brassinosteroid in shoots is required for fruit development in tomato [37]. Two genes were cytochrome P450 (*CYP90A1* and *CYP734A1*) related to brassinosteroids. *CYP90A1* is a brassinosteroid biosynthetic cytochrome P450 in *Arabidopsis* and catalyzes C-3 oxidation [38]. The *CYP734A* subfamily has been shown to inactivate brassinosteroid hormones in *Arabidopsis* and tomato [39]. Plants overexpressing *CYP734A1* exhibit severe dwarfism [40]. The expression of *CYP734A* appears to be up-regulated by exogenous brassinosteroids. In this study, *CYP90A1* and *CYP734A1* were upregulated at 20 DAB in ‘GH81S’ and at 30 DAB in ‘GH59B.’ The expression of *CYP734A1* might be regulated by the synthesize of brassinosteroid induced by *CYP90A1*. The *CYP714* family members were reported involved in GA metabolism. The overexpression of *CYP714A1* and *CYP714A2* in *Arabidopsis* causes dwarfism [41]. Our result showed that one gene of *CYP714A1* (Pbr002890.1) was upregulated at 20 and 30 DAB in ‘GH81S’, which might prevent the fruit development. One gene of WUSCHEL-related homeobox (WOX1) was identified in ‘GH81S’ and ‘GH59B.’ The function of most WOX genes studied so far can be ascribed to the promotion of cell division and/or prevention of premature differentiation [42]. The gene of *WOX1* was upregulated in ‘GH81S’ and ‘GH59B’ at 10 DAB and then the expression decreased at 20 and 30 DAB, suggesting that this gene might promote cell division in the early stage of fruit development.

The transcription factor and protein protein interaction were studied to analyze the regulatory network. Nine *NAC* transcription factors were isolated in this study. *NAC* transcription factors *FEZ* was reported to control the delicately tuned reorientation and timing of cell division in a subset of stem cells in *Arabidopsis*. It is expressed in root cap stem cells, where it promotes periclinal, root cap-forming cell divisions [43]. *JUB1* (*NAC*) was a transcriptional regulator of GA/BR signaling, *JUB1* directly represses the hormone biosynthesis genes *GA3OX1* and *DWARF4* (*CYP90*) [44]. In the present work, the expression of nine NAC transcription factors was consistent with *GA2OX1* and *CKX7*, suggesting that these transcription factors might be related with gibberellin and cytokinin. *ERF* family are conservatively widespread in the plant kingdom involved in the control of primary and secondary metabolism, growth and developmental programs [45]. In this study, Four *ERF* transcription factors were isolated and the expression profile was consistent with *ACO* and *ACS*, suggesting that the ethylene might modulate fruit cell division. *MYB* transcription factors are widely involved in many biological processes, such as the regulation of plant secondary metabolism and the response of hormones and stress [46]. Three *MYB* transcription factors were identified in our result and were annotated to *MYB7* and *MYB8* in *Malus*. The function of these *MYBs* was not clear, which might be related with the development of pear fruit. Overexpression of *GLKs* (GOLDEN2-LIKE) enhances chloroplast development and nutritional quality in tomato [47]. In *Pyrus*, one gene of *GLK2* was upregulated in both of ‘GH59B’ and ‘GH81S,’ which might be related with the fruit development. In the PPI network, CKX7 and ACS were found to interact in the fruit development. CKX7 could degrade the cytokinin and ACS improved ethylene biosynthesis, implying that the cytokinin and ethylene co-regulate the fruit development in pear.

In conclusion, this study confirmed that fruit size difference of *Pyrus pyrifolia* was determined by cell number in young fruit development. To our knowledge, this work is the first study to provide comprehensive sequencing and DEG profiling data for a dynamic view of the transcriptomic variation in pear fruit development. Approximately 797 differentially expressed genes were isolated from the transcriptome in 30 days after blossom, offering new insights into the molecular mechanisms underlying fruit development. Gibberellin and cytokinin related genes were identified that might play important roles in cell division. Our findings provided a relatively complete molecular platform for future studies on the difference of fruit size in pear.

## 4. Material and Methods

### 4.1. Plant Materials

Two pear accessions of ‘GH81S’ and ‘GH59B’ were selected at the experimental farm of Shanghai Academy of Agricultural Sciences in Zhuanhang Town (Shanghai, China) and both of them were crosses between ‘Gold Nijiss Qiki’ (*P. pyrifolia*) and ‘Housui’ (*P. pyrifolia*). The pear trees were 10 years old and considered to be in the adult phase. The phenophase of two pear accessions were similar and the bloom period was in 5 April 2016. We sampled at 10 days after blossom (15 April 2016, 10 DAB), 20 days after blossom (25 April 2016, 20 DAB), 30 days after blossom (5 May 2016, 30 DAB) and 40 days after blossom (15 May 2016, 40 DAB). The mature fruits were collected at 130 days after blossom (15 August 2016, 130 DAB). At each sampling points, eight fruits were collected in one biological replicate and used for RNA extraction. The RNA of three biological replications were pooled by equal quantities (5 μg/replication) to establish one library. Six libraries in two pear accessions at 10, 20 and 30 DAB were used for RNA-seq.

### 4.2. Histological Sections of Young Fruit

The paraffin sections were used to conduct the histological observation. Fruit samples of ‘GH81S’ and ‘GH59B’ were collected at 10, 20, 30, 40 and 130 DAB, immediately fixed in formaldehyde-acetic acid-alcohol fixative and stored at 4 °C. The flesh tissue was dehydrated in a graded ethanol series (70%, 80%, 90%, 100%, 45 min for each concentration) and cleared with xylene for 1 h and then embedded in paraffin. The paraffin block was sliced into thin slices by the microtome of Leica RM2265 (Leica, Solms, Germany). The slices were dried and rehydrated on glass substrates and then stained with safranin (1%, *w*/*w*) for 1 h. After washing with water and ethanol, the slices were stained with fast green (1%, *w*/*w*) for 30 s. The anatomical images were observed using a microscopic imaging system of Eclipse Ti-S (Nikon, Tokyo, Japan).

### 4.3. RNA Extraction and RNA-Seq

Total RNA was extracted using the CTAB method [48]. RNA purity and concentration were assessed using the NanoDrop 2000 (Thermo, Waltham, MA, USA). Genomic DNA was digested with DNase I. The cDNA libraries were constructed with approximately 5 μg of RNA for each sample using the NEBNext Ultra RNA Library Prep Kit (NEB Inc., San Diego, SF, USA) according to the manufacturer’s instructions and index codes were added to attribute sequences to each sample. To select cDNA fragments of preferentially 250–300 bp in length, the library fragments were purified using the AMPure XP System (Beckman Coulter, Los Angeles, CA, USA). Subsequently, 3 μL of USER Enzyme (NEB) was used with size-selected, adaptor-ligated cDNA at 37 °C for 15 min. Then PCR (20 μL total volume) was performed using 10 μL of Q5 Hot Start HiFi PCR Master Mix (M0543, NEB Inc., San Diego, CA, USA), 1 μL of Universal PCR primers, 1 μL of Index (X) Primer, 2 μL of cDNA and 7 μL of RNase-free water. The PCR products were purified (AMPure XP system) and library quality was assessed on the Agilent Bioanalyzer 2100 system (Agilent, Santa Clara, CA, USA). Each library (approximately 10 ng) was used for Paired-End sequencing using Illumina HiSeq™ 4000 (Illumina, San Diego, CA, USA). Raw sequence data in the FASTQ format were filtered to remove reads containing adaptors, reads with more than 5% unknown nucleotides and low-quality reads with more than 20% bases of quality value ≤10. Only clean reads were used in the following analysis. The clean reads data have been deposited in the NCBI Sequence Read Archive (http://www.ncbi.nlm.nih.gov/sra/) and the SRA accession number is SRP150403.

### 4.4. Identification of Differentially Expressed Genes (DEGs)

Clean reads were mapped to the *Pyrus* reference genome (NCBI, AJSU00000000) using TopHat and cufflinks software (Hopkins, Baltimore, MD, USA), allowing mismatches of no more than two bases. The unique mapped reads were used in subsequent analyses. The gene expression level was calculated using the method of reads per kb per Million reads (RPKM). Baggerly’s test and the false discovery rate (FDR) with a significance level of ≤0.001 and the absolute value of Log_2_Ratio ≥ 1 were set as the threshold to determine the significance of the gene expression difference. KEGG pathway analysis was performed by first mapping all DEGs (Differential Expressed Genes) to KEGG terms in the database (https://www.genome.jp/kegg/pathway.html), calculating the gene numbers for every term and subsequently using the hypergeometric test to identify significantly enriched KEGG terms in the input list of DEGs.

### 4.5. Expression Analysis of Quantitative Real-Time PCR

Total RNA was extracted from the fruits at four stages, 10, 20, 30 and 40 DAB, using three biological replicates. First-strand cDNA was synthesized from 1 μg of total RNA using the PrimerScript RT reagent Kit with gDNA Eraser (RR047, TaKaRa, Osaka, Japan), diluted 10 times with H_2_O and subsequently used as templates for Q-PCR assays. The Q-PCR mixture (15 μL total volume) contained 7.5 μL of SYBR Premix ExTaq (RR820, Takara), 0.5 μL of each primer (10 μM), 2 μL of cDNA and 4.5 μL of RNase-free water. The reactions were performed on a LightCycler480 instrument (Roche, Basel, Switzerland) according to the manufacturer’s instructions. The two-step Q-PCR program was initiated at 95 °C for 30 s, followed by 40 cycles at 95 °C for 5 s and 60 °C for 20 s. Template-less controls for each primer pair were included in each run. The specificity of the Q-PCR primers was confirmed using a melting curve and the Q-PCR products were sequenced (Table 2). Expression was calculated as 2^−ΔΔCt^ and normalized to that of the actin gene (JN684184) [49] and UBI (AF179386) [50] and the data were managed using the Data Processing System (DPS, v. 7.05).

## Figures and Tables

**Table 1:** Statistics of the reads in the present study.

Samples	Raw Reads	Total Clean Reads	Total Mapped Reads	Q20	Q30
GH81S-10 DAB	47,463,332	43,555,990	70.40%	98.31%	95.38%
GH81S-20 DAB	50,756,314	46,213,290	69.20%	98.25%	95.26%
GH81S-30 DAB	52,395,106	46,794,198	68.70%	98.40%	95.55%
GH59B-10 DAB	50,756,688	46,472,938	69.80%	98.35%	95.47%
GH59B-20 DAB	50,018,632	45,642,660	69.70%	98.27%	95.31%
GH59B-30 DAB	50,757,620	45,838,906	69.00%	98.10%	94.89%

**Table 2:** The primers of Q-PCR in present study.

Primer	Sequence	Tm (°C)	Length (bp)	Accession Number	PCR Efficiency%
Q-ZOG1-F	CCACCTCAACCAACTCCTACAC	58.6	97	XM_018648918	107
Q-ZOG1-R	CTTAACTTGGCGGTTGTGAGTG	60.1			
Q-GA2OX1-F	GCAGATAACAGGCTTGGACACTT	60.4	123	XM_009356581.2	108
Q-GA2OX1-R	GATTTCCGAGTACCGAGATTGAA	60.3			
Q-CKX7-F	GAGATTTGTGGAGCGGAAGAC	58.8	188	XM_009336405.2	109
Q-CKX7-R	CCAGTAGAAACTAATCAAGCCAATA	57.7			
Q-CKX6-F	AAAATCTGCTTACGACCCCTTGG	63.8	124	XM_009369812.2	96
Q-CKX6-R	ACATGCCTTTAGGGCCTCTTCTT	62.8			
Q-ACS-F	TGTCTCCTCATACACCGATACCC	60.5	171	XM_009365119.2	102
Q-ACS-R	GAAAGAAGGTATCCACCACTCAA	57.9			
Q-ACO-F	TCCCAGTTGTTGACTTGAGCCT	61.2	194	NM_001302321.1	93
Q-ACO-R	CATTTCCTTAAACCTTTGCTCCA	60.7			
Q-NAC25-F	TTCTACCCTAATCCTGCACTTCT	57.3	118	XM_009381159.2	108
Q-NAC25-R	CATCTTAAACCCACCATCCAAA	59.3			
Q-WOX1-F	TGTTACTGGGAGGCGTTTAGATT	60.2	119	XM_009361806	105
Q-WOX1-R	AATACAATGGCGCTTATACAAGTC	58.1			
Q-actin-F	CCATCCAGGCTGTTCTCTC	54.7	139	JN684184	100
Q-actin-R	GCAAGGTCCAGACGAAGG	55.7			
Q-UBI-F	ACCCTCGCCGACTACAAC	55.3	198	XM_009368893.2	94
Q-UBI-R	ACTCCTTCCGCAGCCTCT	55.4			

## Supplementary Materials

Supplementary materials can be found at http://www.mdpi.com/1422-0067/19/8/2342/s1.
