# Supplementary figures and images for: Comparative Analysis of Transcriptomes to Identify Genes Associated with Fruit Size in the Early Stage of Fruit Development in Pyrus pyrifolia
Source: Int J Mol Sci. 2018 Aug 9;19(8):2342. doi: 10.3390/ijms19082342 (PMC6122012; doi:10.3390/ijms19082342)

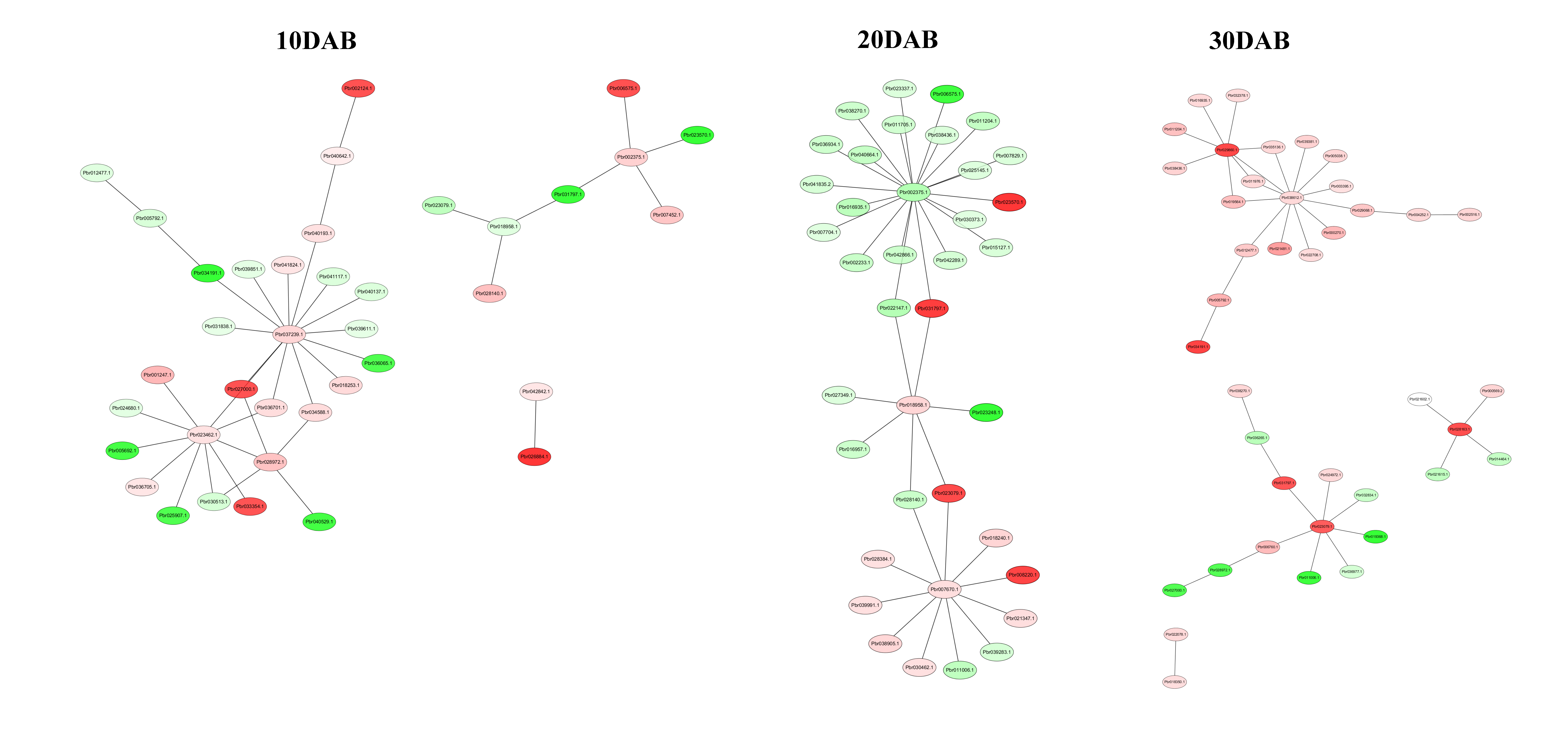

Supplement: Supplementary file 1 [file ijms-19-02342-s001.zip › Fig S1.png]
